# A Printed Multicomponent Paper Sensor for Bacterial Detection

**DOI:** 10.1038/s41598-017-12549-3

**Published:** 2017-09-26

**Authors:** M. Monsur Ali, Christine L. Brown, Sana Jahanshahi-Anbuhi, Balamurali Kannan, Yingfu Li, Carlos D. M. Filipe, John D. Brennan

**Affiliations:** 1 Biointerfaces Institute, McMaster University, 1280 Main St W, Hamilton, Ontario, L8S 0A3 Canada; 2 Department of Chemical Engineering, McMaster University, 1280 Main St W, Hamilton, Ontario, L8S 4L7 Canada; 3 Department of Biochemistry and Biomedical Sciences, McMaster University, 1280 Main St. W, Hamilton, ON L8S 4K1 Canada

## Abstract

We present a simple all-in-one paper-based sensor for *E. coli* detection using a composite ink made of a fluorogenic DNAzyme probe for bacterial recognition and signal generation, lysozyme that lyses whole bacterial cells, and pullulan/trehalose sugars that stabilize printed bioactive molecules. The paper sensor is capable of producing a fluorescence signal as a readout within 5 minutes upon contacting *E. coli*, can achieve a limit of detection of 100 cells/mL, in a variety of sample matrixes, without sample enrichment, and remains stable for at least 6 months when stored at ambient temperature. Therefore, this simple paper sensor provides rapid bacterial testing on site, and can be shipped and stored under ambient conditions to benefit users living in resource-limited regions.

## Introduction

There has been a significant effort over the past decade to develop low-lost, user-friendly point-of-care (POC) diagnostics using paper as an inexpensive and disposable substrate^1–10^. Paper is widely available and can be modified by printing a wide range of reagents, including diverse biorecognition elements that include proteins, antibodies, nucleic acids, various amplification systems (such as polymerase chain reaction and rolling circle amplification) and assay reagents to produce colorimetric, fluorimetric or electrochemical outputs for a variety of analytes^2,11^. The availability of high throughput dispensers and printers as well as advanced microfabrication techniques allows accurate design of small diagnostic devices with minute amounts of reagents that require microliter sample volumes, and provides a facile route to scalable manufacturing of such devices^12,13^. One area where paper-based sensors can make a substantial impact is on the early detection of infectious organisms, either in the environment or in clinical samples. Bacterial infection linked to contaminated food and water causes millions of deaths annually, particularly in the developing world where routine testing is currently not possible^14–20^. Current tests are either too slow (plate culture methods^21^), too expensive and/or complicated (PCR, enzyme linked immunoassays^21,22^), or too insensitive. For example, lateral flow immunoassays typically have detection limits of ~10^5^–10^7^ cells/mL^22–24^ while paper-based assays detecting intracellular enzymes have detection limits of ~10^4^ cells/mL^25^, requiring sample pre-concentration or enrichment *via* culturing prior to testing. Therefore, there remains a need to develop a rapid, sensitive, portable test for infectious organisms, which can be used on unenriched samples, be easily produced using printing technology and that can retain full functionality under ambient storage conditions for an extended period of time.

**Figure 1:**
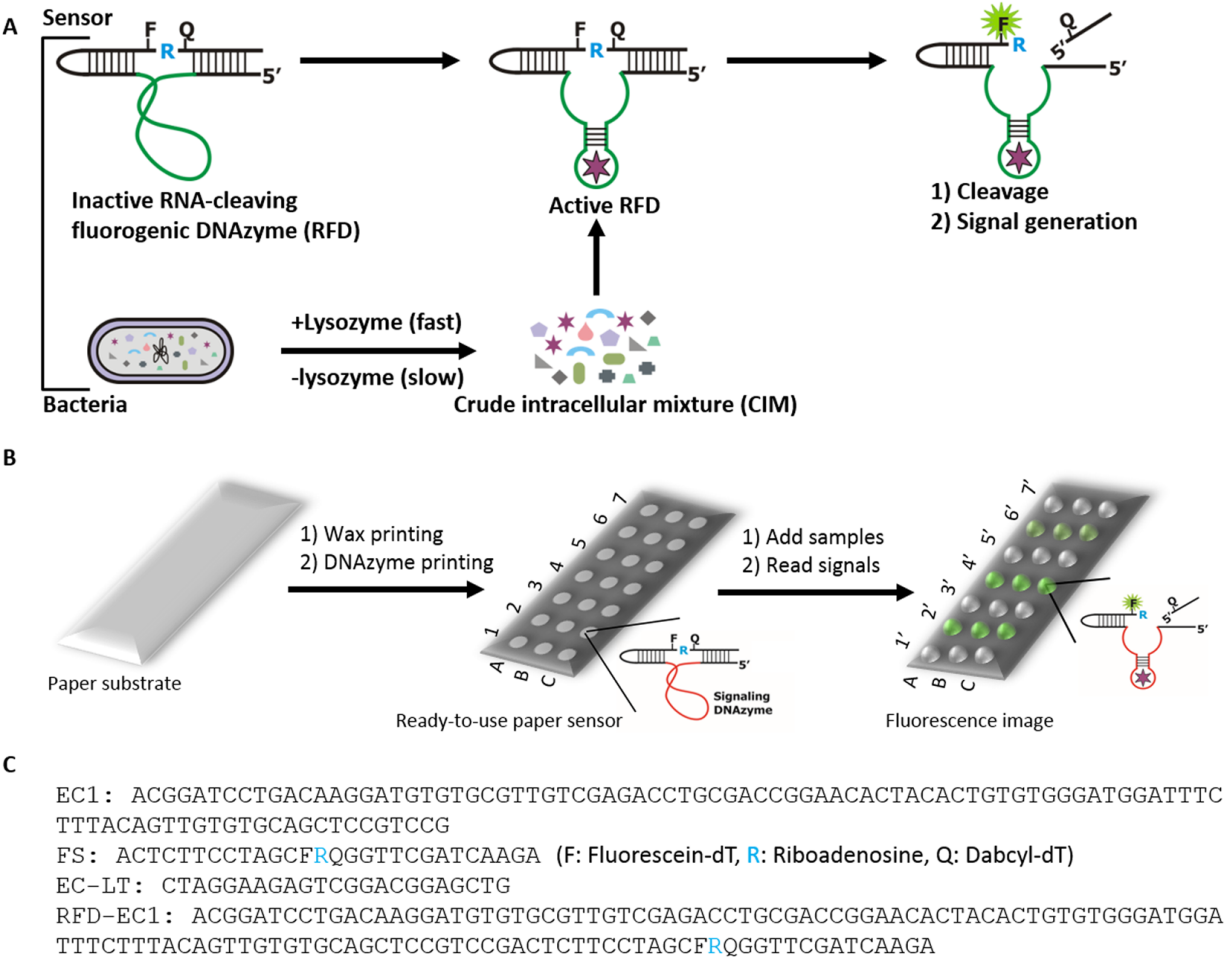
Schematic illustration of the mode of action of the DNAzyme and envisioned paper device with the DNAzyme sensor. (**A**) The inactive DNAzyme (upper panel, left image) becomes activated upon interaction with the target(s) that are released from the bacteria (upper panel, middle image). The active DNAzyme then cleaves the fluorogenic substrate to produce fluorescent signal (upper panel, right image). (**B**) A paper substrate (left image) is wax-printed to provide microzones (white zones) using a printer. The DNAzyme sensor mixed with a pullulan + trehalose + lysozyme solution is printed in each microzone and air-dried. This dried paper device is now ready-to-use for bacterial assays (middle image). The numbers (1–7) represent the sample identity whereas the letters A, B and C denote triplicates of the same experiment. Samples are applied to test zones and allowed to react. If a sample contains the target bacteria (*E. coli* in this case), the DNAzyme cleaves the fluorogenic substrate and produces a fluorescent signal, which is captured using a fluorescent scanner and analysed using suitable software (far right image). (**C**) DNA oligonucleotides sequences. EC represents the DNAzyme sequence for *E. coli*; FS is the fluorogenic substrate; EC-LT is the template; and RFD-EC1 is the complete DNAzyme sequence including EC and FS that is used in the experiments.

We have recently developed a versatile *in vit*r*o* selection technique that can be used to isolate highly selective RNA-cleaving fluorogenic DNAzymes (RFDs) for bacterial pathogens^26,27^. The working principle of the RFDs is shown in Fig. 1A. The inactive form of the RFD is allosterically converted into an active form upon interaction with the target molecule(s) present in the crude intracellular mixture (CIM) of the specific bacterium to catalyze the cleavage of the fluorogenic substrate and thereby produce a fluorescence signal. Using *Escherichia coli* (*E. coli*) as a model bacterium, we have shown that these RFDs can be used to develop simple solution-based assays for bacteria^26,28–30^. Since these RFDs are solely made of synthetic DNAs, they are more stable than their protein and RNA counterparts, which should make them suitable for developing paper-based POC diagnostics for the detection of bacteria in low resource settings.

Herein we describe, for the first time, a paper-based DNAzyme sensor for rapid and sensitive detection of *E. coli*. As shown in Fig. 1B, this sensor can be fabricated on a paper substrate by a simple two-step process involving wax printing of microzones followed by ink-jet printing of a DNAzyme-loaded ink into the microzones. The resultant sensor is capable of producing an increase in fluorescence intensity upon DNAzyme cleavage of its fluorogenic substrate (see sequences in Fig. 1B) if the test sample contains *E. coli*. The device can be fabricated to contain many test zones to examine multiple samples in multiple replicates.

## Results

### Fabrication of Printed DNAzyme-Based Sensor

The paper-based assay is based on a previously reported RNA-cleaving fluorogenic DNAzyme probe for *E. coli*, known as RFD-EC1^26^. Given that the assay relies on fluorescence signal generation, it was critical to select a suitable paper substrate with low background fluorescence. After testing several types of paper, we determined that nitrocellulose paper (NCP, nitrocellulose membrane backed with a thin plastic layer on one side) and Whatman #1 filter paper had the lowest background fluorescence (see Fig. S1A). We selected NCP for this study because the nitrocellulose paper prevents diffusion and leaching of aqueous samples through the material, which helps to retain the sample within the microzones, allowing longer reaction times to produce higher signals.

**Figure 2:**
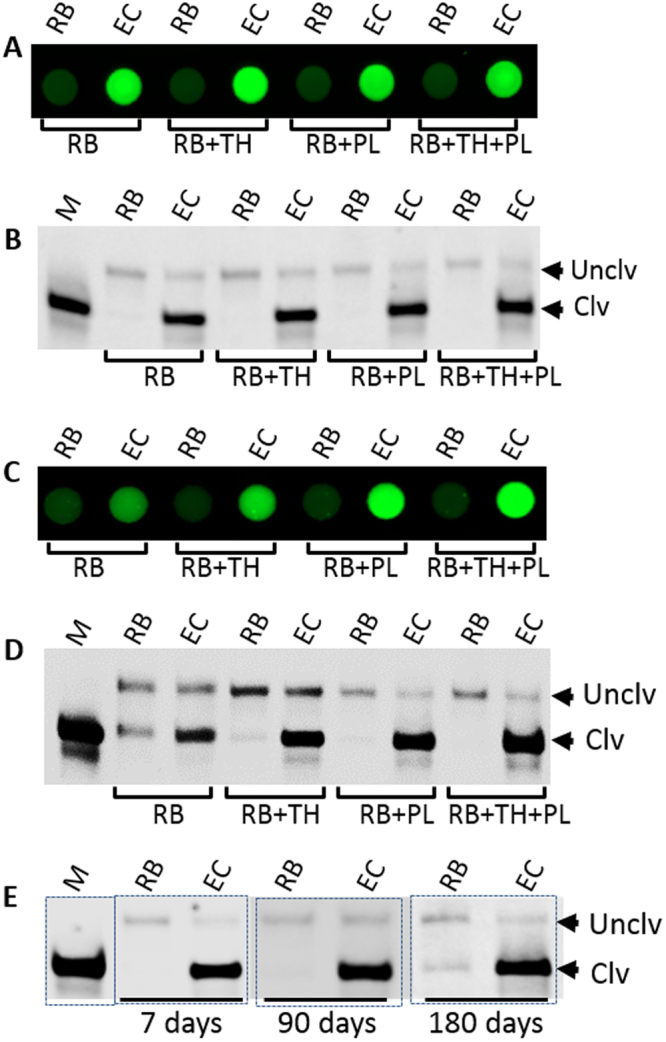
Optimization of the conditions for immobilizing the DNAzyme sensor and results of long-term stability tests. (**A**) Fluorescent image after the cleavage reaction of the DNAzyme 24 h after printing. The DNAzyme probe was printed with 4 different conditions: (1) with reaction buffer (RB) alone, (2) with RB including trehalose (TH), (3) with RB including pullulan (PL), and (4) RB including TH and PL. EC stands for *E. coli*. (**B**) Denaturing gel image of the reaction mixtures of the experiments in A (Full gel image is provided in the ESI, Fig. S2A, with the dashed line box delineating the section used to produce Fig. 2B.) (**C**) Fluorescent image after the cleavage reaction of the DNAzyme after 7 days of storage using the same printed components described in A. (**D**) Denaturing gel image of the reaction mixtures of the experiments in C (Full gel image is provided in the ESI, Fig. S2B with dashed line box delineating the section used to produce Fig. 2D.) (**E**) Stability test of the DNAzyme after printing on paper using the optimized conditions. The image is a representative denaturing gel image of the cleavage reaction on paper (individual gel images were obtained at each storage time; the original gel images are provided in the ESI Fig. S2B–D. The sections used to construct composite Fig. 2E are indicated with dashed boxes).

To produce a multiplexed sensor, the NCP was modified by wax printing followed by heating to produce individual microzones (~4 mm diameter) surrounded by a hydrophobic barrier (Fig. S1B). Printing of the DNAzyme probe was investigated by testing a trehalose-modified pullulan ink, which was selected based on a previously reported screen of various pullulan ink formulations^13,31^. Both sucrose and trehalose have previously been utilized as stabilizing agents for nucleic acids and proteins^32–35^. However, trehalose, which is a biostabilizing osmolyte, was more compatible with the formulation of a biocompatible and printable pullulan-based bio-ink. RFD-EC1 was printed into the microzones with 4 different conditions: 1) reaction buffer alone (RB), 2) RB including trehalose (RB + TH), 3) RB containing pullulan (RB + PL) and 4) a mixture of RB, PL and TH (RB + PL + TH, see experimental section for details). It is interesting to note that, upon drying, the microzones that contained pullulan (Fig. S1C, labeled as RB + PL, RB + PL + TH) turned into transparent zones while RB and TH without pullulan did not change the physical appearance of the microzones (Fig. S1C, labeled as RB, RB + TH), suggesting that the pullulan inks fully penetrated the membrane and produced a clear zone owing to refractive index matching. Cleavage reactions on the microzones, which were induced by adding either RB alone or the crude intracellular mixture of *E. coli* (CIM-EC), showed that the fluorescence signal was much stronger in the microzones that were treated with CIM-EC compared to those treated with RB (Fig. 2A), demonstrating the efficient cleavage of the substrate by the DNAzyme and the concomitant increase in fluorescence in the presence of the target.

To verify that the signal arose from cleavage of the substrate, we analyzed the reaction mixtures collected from each microzone by 10% denaturing polyacrylamide gel electrophoresis (dPAGE). The gel images showed that the cleaved products appeared only with the DNAzyme samples that were treated with CIM-EC (Fig. 2B, see images of all full gels in the Supporting Information, Fig. S2), confirming that the fluorescence signals resulted from cleavage of the substrate by the DNAzyme in the presence of CIM-EC. At early storage times (24 hours) the inclusion of polysaccharides to the ink formulation had no impact the signal level (Fig. 2A, Fig. S2A for quantitative analysis). However, at longer storage times (7 days) the DNAzyme printed with reaction buffer (RB) alone or RB with trehalose produced a lower signal, which was due to background cleavage of the substrate (as confirmed by dPAGE analysis), while the use of polysaccharide-based inks produced substantially higher signal levels owing to lower background cleavage of the substrate (Fig. 2C,D), with the PL + TH formulation producing the highest signal and the best cleavage (see Fig. S3 for quantitative analysis). Long-term stability studies of the printed DNAzyme with the TH + PL formulation showed that the immobilized DNAzyme probe remained stable for at least 6 months when stored at ambient temperature (Fig. 2E), which should allow transportation and storage to remote locations for use in resource limited settings. Therefore, we chose the PL + TH formulation for all subsequent experiments.

### Analytical Performance of the Paper Sensor

RFD-EC1 was isolated by *in vitro* selection using a crude extracellular mixture of *E. coli* (CEM-EC) as a complex target after culturing *E. coli* in culture media^26^. However, our later studies indicated that cellular lysates (CL, excluding the culture media) produced a higher signal and improved the detection sensitivity^30^. Therefore, efficient release of the crude intracellular mixture (CIM) should shorten the detection time and achieve better sensitivity for whole cell analysis. We recently demonstrated the ability to lyse *E. coli* cells directly on a paper device by printing of cell lysis reagents such as lysozyme onto the device^36^. We therefore included lysozyme (100 µg per microzone) as a component in the TL + PL bioink and evaluated the sensitivity of the sensor relative to that without lysozyme being present. Bacteria that were lysed by heating were also included as a positive control.

**Figure 3:**
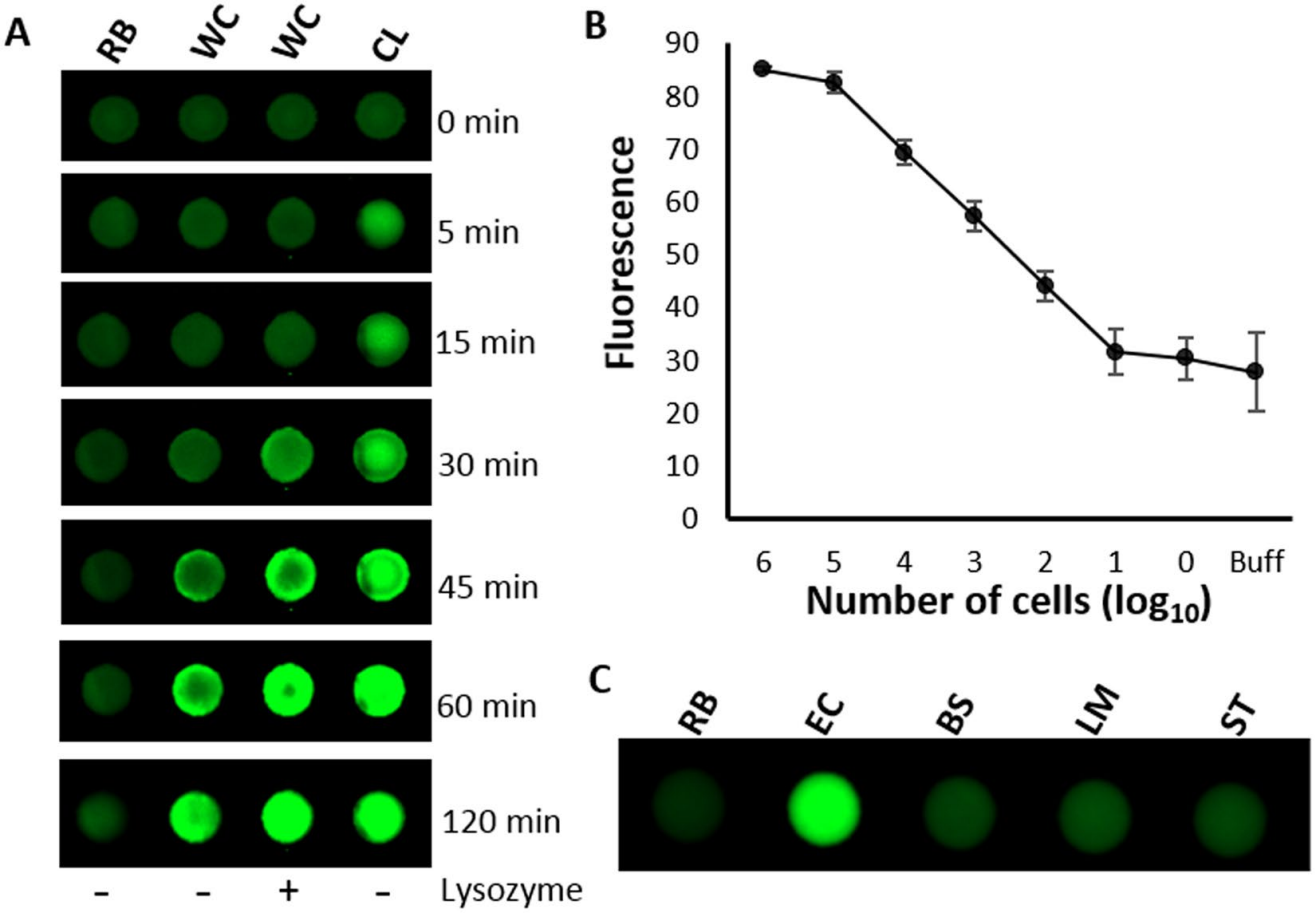
(**A**) Assessment of the effect of lysozyme in the printed bio-ink on the fluorescence signal. The DNAzyme with pullulan and trehalose was mixed without or with lysozyme and printed on the paper (the lane shown as − and + lysozyme at the bottom). In the first zone only buffer was added, in the second and third zone whole cells (WC) were added. In the fourth zone cell lysate (CL) was added. The paper was scanned for fluorescence at different time points as indicated. (**B**) Detection limit was investigated with different numbers of *E. coli* cells without lysis (WC) and after lysis (CL). Details on obtaining different number of cells, cleavage reactions and analysis are provided in the experimental section. (**C**) Selectivity test using non-target gram positive and gram negative bacteria.

As shown in Fig. 3A, the inclusion of lysozyme to a 8% (w/v) pullulan bioink produced a significant increase in the rate of signal development relative to sensors without lysozyme included, which allowed generation of a large signal within 30 min (compare lanes 2 and 3 in Fig. 3A) using whole cells. For comparison, a positive control comprised of a cell lysate produced a detectable signal within 5 minutes (Fig. 3A, labeled with CL), and consistently higher signal levels relative to whole cell samples tested on lysozyme-containing sensors, suggesting incomplete lysis of the whole cells. However, the inclusion of lysozyme in the bioink did provide a 2–3× improvement in signal intensity (see Fig. S4 in the Supporting Material) at early reaction times, and thus allowed more rapid detection. We noted, however, that both lysed and unlysed whole cell samples showed identical maximum signals after 90 min (see Fig. S5), indicating that even untreated whole cells underwent lysis to release the intracellular target within this time frame. Given this finding, it is unlikely that RFD-EC1 can discriminate live vs. dead cells, since both will eventually release intracellular target(s) although live cells release target at a slower rate. In such as case it would be necessary to implement a culture step to discriminate live from dead cells – such a step is a regulatory requirement when testing drinking water for bacterial contamination^37^. Furthermore, while positive controls comprised of heat induced cell lysates were able to generate signals more quickly, it required treatment at 90 °C, which is not ideal for field applications. Therefore, all further work involved directly applying whole cell samples to the microzones. The results shown in Fig. 3B indicate that under these conditions, the paper device can detect 100 cells in 90 minutes (with or without lysing agent present).

Previous reports on RFD-EC1 have demonstrated that the DNAzyme is very specific for *E. coli* ^26,38,39^. As shown in Fig. 3C, the paper sensor maintained the specificity for *E. coli* over other bacteria such as *Bacillus subtilis* (BS), *Listeria monocytogenes* (LM) and *Salmonella typhimurium* (ST), (see Supporting Fig. S6 for quantitative data on selectivity).

**Figure 4:**
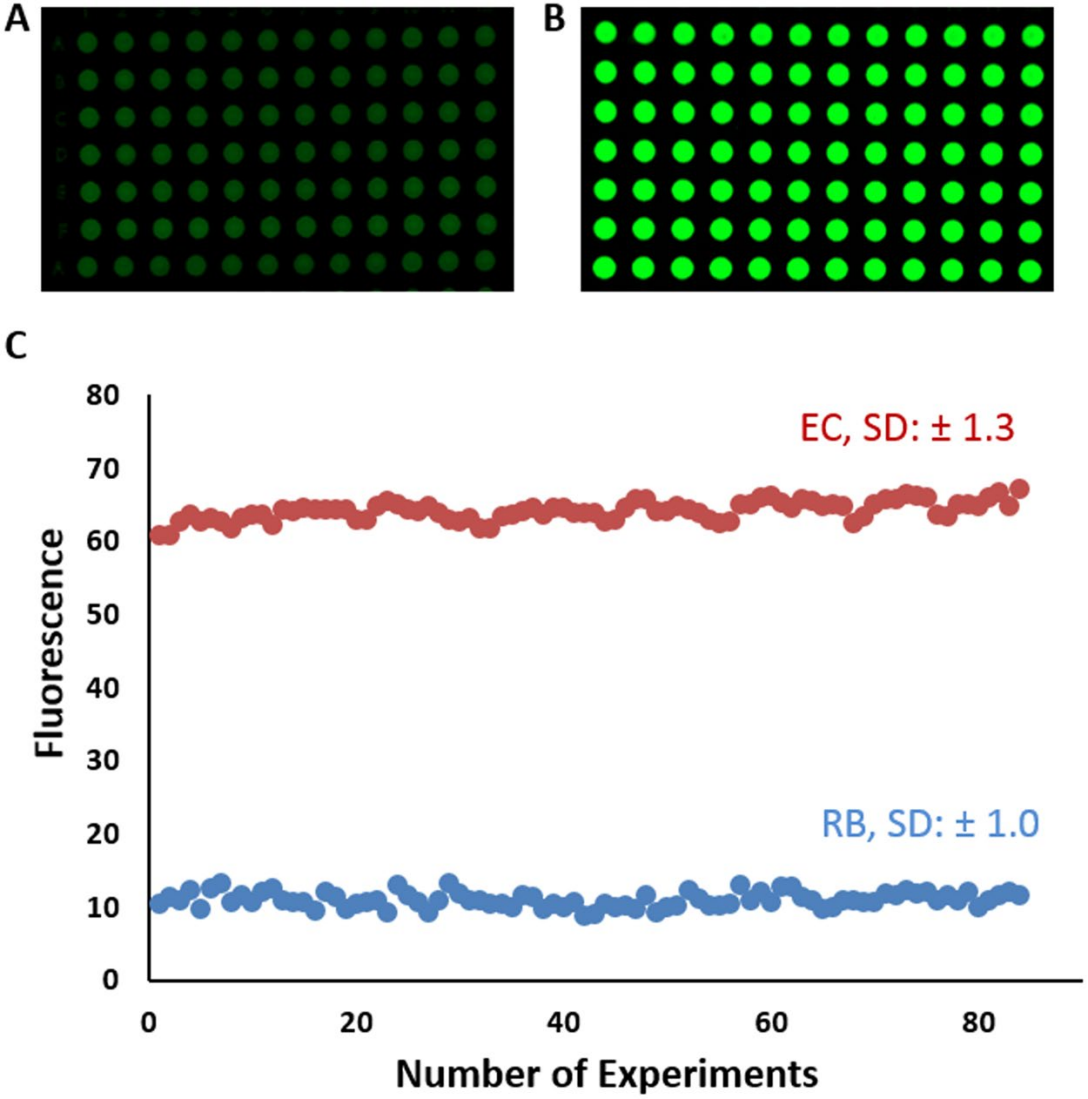
High throughput printing of DNAzyme and evaluation of signal levels upon addition of target sample. The DNAzyme probe was printed onto two paper devices using a Biodot printer, followed by drying. Fluorescence signaling was evaluated by adding the reaction buffer (**A**) and *E. coli* cell lysate (**B**) using the Biodot printer to ensure consistent sample volumes were dispensed. After the cleavage reaction, the fluorescent signal was quantified and plotted (**C**). The standard deviation (SD) was calculated from 84 individual experiments on paper.

We finally tested the feasibility of mass production of detection assays by printing assay spots in a 96-well arrangement. We used the bioink formulation consisting of 8% (w/w) pullulan with 0.25 M (w/v)% trehalose^13^, and printed the material using a Biodot printer at a rate of 100 wells per minute. As shown in Fig. 4, the printed tests produce a highly reproducible fluorescence output with very low error between the different DNAzyme sensors that were exposed to the cell lysate of *E. coli*. These results demonstrate that the paper based DNAzyme microzone assay for bacterial detection can be produced rapidly in a manner compatible with scalable automated manufacturing.

### Performance in Spiked Beverages

**Figure 5:**
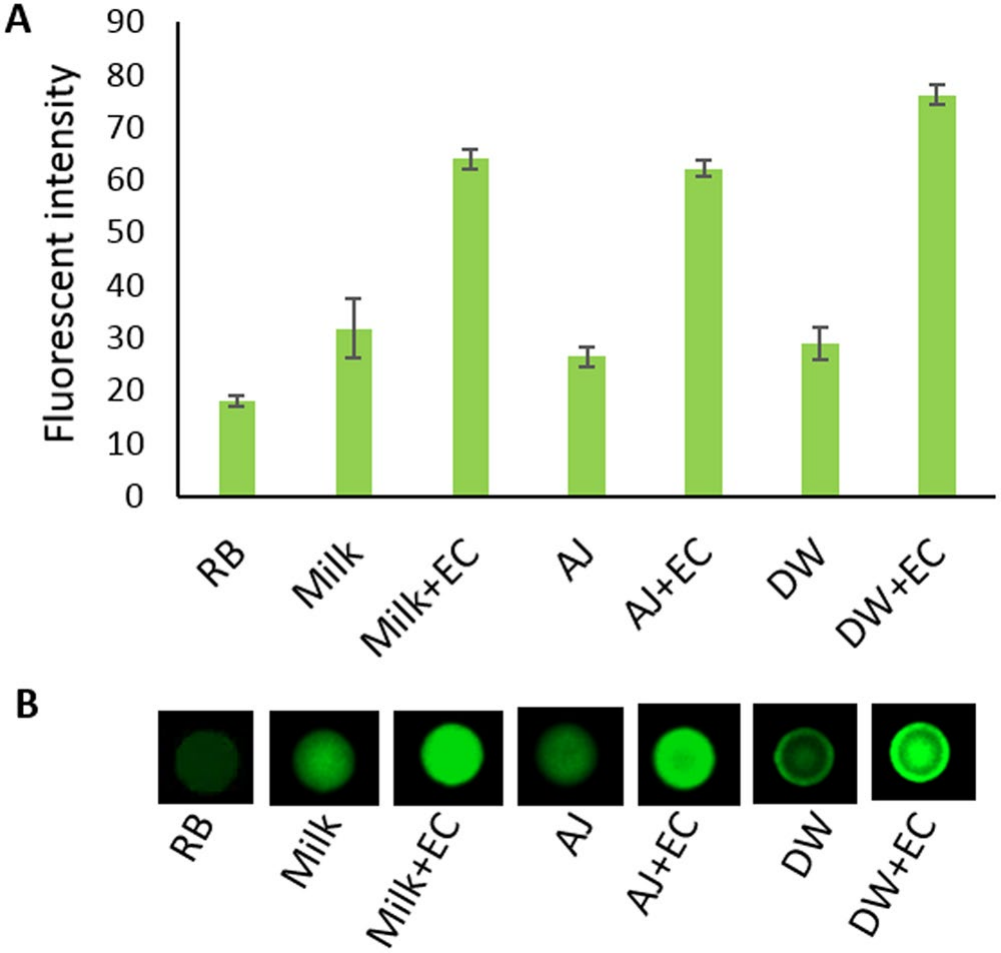
Performance of the RFD-EC1 DNAzyme in milk, apple juice and drinking water samples. (**A**) Fluorescence signal of the DNAzyme in reaction buffer alone (RB), diluted milk alone (milk), diluted milk spiked with *E.coli* (milk + EC), diluted apple juice alone (AJ), diluted apple juice spiked with *E. coli* (AJ + EC), drinking water alone (DW), drinking water spiked with *E. coli* (DW + EC). The error bars are based on the standard deviation of triplicate experiments. (**B**) A representative fluorescent image of each of the experiments described in part A.

In our previous reports, it was shown that the RFD-EC1 DNAzyme performs well in biological samples such as whole blood, milk and lake water that were spiked with *E. coli* ^38,40^. However, the study with whole blood utilized a droplet microfluidics platform, while the study with milk, juice and lake water utilized a colorimetric signaling format. Since our current study is fluorescence-based and uses a one-step “mix and read” assay, we evaluated its performance using common beverages such as milk, juice and drinking water. Each of the samples was spiked with *E. coli*. at a level of 10^7^ cfu/mL and tested for signal production performance. The performance in drinking water was found to be excellent (Fig. 5A,B, labelled as DW, DW + EC). As milk and juice are more complex samples and their autofluorescence and pH may impact on the DNAzyme performance, we first tested the autofuorescence at different dilutions of each sample (Supporting Fig. S7) after mixing with the reaction buffer (RB). As expected, although milk did not change the pH, it produced high background fluorescence when diluted 2-fold or 4-fold. However, when diluted 8-fold (12.5% solution), the sample produce a low background fluorescence signal, thus 12.5% milk was used for the signal generation test. On the other hand, apple juice did not produce a high background signal, but was sufficiently acidic that even a 2-fold dilution did not raise the pH above 6.0. Therefore, at 4-fold dilution (25% solution) of juice was used to evaluate the DNAzyme cleavage and signal generation. The results depicted in Fig. 5A,B show that, in both samples, the DNAzyme performed well, generating at least a 2-fold increase in signal above background.

### Nuclease degradation test

We previously reported on the stability of functional nucleic acids (DNA, RNA) against nucleases when entrapped in pullulan films and stored under dry conditions^39^, but were further interested to determine if the printed DNAzyme was protected against nucleases. In this case, we included the nuclease (RNase I) directly in the DNAzyme-pullulan solution prior to printing, and then printed the solution immediately onto NCP. After air drying overnight, reaction buffer was added (with no *E. coli* present) and scanned for fluorescence intensity. It was observed that there was no fluorescence enhancement compared to the control where the DNAzyme was printed with pullulan alone without nuclease (Supporting Fig. S8 in the ESI). Hence, the RNA linkage in the DNAzyme substrate remained intact even in the presence of nuclease.

## Discussion

The present study describes a simple method to produce printed polysaccharide/lysozyme/DNAzyme-containing paper sensors for detection of unenriched bacteria. In comparison to the solution-phase test^26,30^, the paper sensor not only has very similar performance characteristics but also offers unmatched advantages that include a simpler testing procedure, better product stability and no need for cold shipping and storage. These benefits are highly desirable for the design of simple but effective tests for field applications, particularly for resource-limited regions.

The inclusion of sugar molecules - pullulan and trehalose - in the bioink formulation is crucial for high functionally and stability of the printed paper sensor. As we have shown previously, these saccharides are able to prevent the denaturation of enzymes, degradation of labile small molecules, and hydrolysis of RNA species by auto-cleavage and RNase-induced degradation^13,31,39^.

The addition of lysozyme in the bioink adds another advantage of the bacterial paper sensor as it helps lyse the whole cells and more rapidly release target molecules to activate the DNAzyme on the sensor. Although the use of pre-lysed cell samples result in a shorter detection time, the heat step that is needed for making such samples makes it inconvenient for an on-site test. On the other hand, the paper sensor without printed lysozyme is also effective, but requires a much longer reaction time. Therefore, the inclusion of the lysozyme offers a balanced approach with a relatively short test time and simpler testing procedure, and allows testing of untreated samples simply by adding the sample to the paper microzone.

The printed DNAzyme sensor is capable of detecting 100 *E. coli* cells in a 10 µL sample volume applied to the paper sensor, which correlates to 10^4^ cells/mL for water samples, and a somewhat higher value of 10^7^ cells/mL for milk or juice samples. The detection sensitivity is better than lateral flow immunoassays that typically afford detection limits of ~10^5^–10^7^ cells/mL in water^22–24^, and on par with other paper-based bacterial sensors^25^. To improve sensitivity, a simple filtration step could be incorporated to reduce the total volume of the sample and increase bacterial concentration^25^. The detection sensitivity could also be further enhanced by incorporating an additional signal amplification step. For example, we have recently shown that the cleavage product of the *E. coli*-sensing DNAzyme can be subjected to DNA amplification via an isothermal DNA amplification technique known as “rolling circle amplification”, significantly improving detection limits^41^.

Although the current work used *E. coli* as the target of detection, the same approach can be easily expanded for detection of a range of additional bacterial pathogens through the use of specific DNAzyme probes that can be selected from random-sequence DNA libraries by *in vitro* selection^26,27^. Development of such sensors will be reported in future studies.

## Conclusions

In summary, we have developed a simple and robust paper-based mix-and-read assay using a DNAzyme for bacterial detection that avoids the need for sophisticated instrumentation and technical expertise. Using an *E. coli*-specific DNAzyme as a model system, we have shown that the assay is sensitive enough to detect as few as 100 whole *E. coli* cells in a small volume of water. Importantly, this sensing device is stable and functional upon storage for extended periods of time at room temperature. We have also shown that multiple tests can be produced simultaneously and the resultant sensors are capable of generating consistent signals. We believe that our work represents a new direction for developing paper-based point-of-care diagnostics based on integrating advanced DNAzyme assays with modern printing technologies.

## Materials and Methods

### Materials and Reagents

The DNAzyme sequence (EC in Fig. 1B) was purchased from Integrated DNA Technologies (IDT, Coralville, IA, USA) and purified by 10% denaturing polyacrylamide gel electrophoresis (dPAGE). The substrate (FS: in Fig. 1B) was obtained from the W. M. Keck Oligonucleotide Synthesis Facility (Yale University, New Haven, CT, USA), deprotected and purified by 10% dPAGE following a previously reported protocol^24^. T4 DNA ligase (TDL) and T4 polynucleotide kinase (PNK), including their respective buffers, were purchased from Thermo Fermentas (Burlington, ON, Canada). Tryptic Soy Broth (TSB) was acquired from BD Biosciences. Nitrocellulose paper (HF120), and Whatman filter paper #1 and #3 were purchased from GE Healthcare, Canada. Regular printer paper was purchased from Grand & Toy, Canada. Food packaging hard paper was acquired from Cascades, Canada. Plastic backed Whatman #1 was obtained from McMaster University campus store. Unless otherwise noted, all other reagents were purchased from Sigma and used without purification.

### Bacterial Cells

The bacterial cells used in this study included *Escherichia coli* K12 (MG1655), *Bacillus subtilis* 168, *Listeria monocytogenes* and *Salmonella typhimurium*, which are regularly maintained in our laboratory.

### Preparation of RFD-EC

The full length DNAzyme sequence including the substrate (RFD-EC in Fig. 1B) was prepared by template-mediated ligation of FS to EC following our previously reported protocol^26^. Briefly, 500 pmol of FS was phosphorylated by treating with 1 mM ATP and 10 U (units) of PNK for 30 min at 37 °C (reaction volume = 100 μL) in 1×PNK buffer A (50 mM Tris–HCl (pH 7.6), 10 mM MgCl_2_, 5 mM DTT, and 0.1 mM spermidine). The reaction was quenched by heating at 90 °C for 5 min followed by the addition of one equivalent each of EC and EC-LT. The reaction mixture was heated at 90 °C for 30 s and cooled to room temperature for 20 min. Next, 20 μL of 10×TDL buffer (Thermo Fermentas: 40 mM Tris–HCl, 10 mM MgCl_2_, 10 mM DTT, and 0.5 mM ATP, pH 7.8) and 4 μL of TDL (5 U/μL) were added and the reaction volume was adjusted to 200 μL with ddH2O. After 2 h of incubation at room temperature (RT), the ligated DNAzyme product (RFD-EC) was purified by 10% dPAGE.

### Microzones Fabrication on Paper

Microzones of ~4 mm diameter were drawn in PowerPoint with a 6 mm inter-microzone distance, aligned in 8 rows and 12 columns (as shown in Fig. S1B). Wax was then printed on various paper substrates using a Xerox Phaser 8560 N wax printer. The wax-printed paper was heated at 120 °C for 2 minutes to melt the wax into the pores of the paper and provide hydrophobic barriers around the microzones.

### Preparation of Crude Intracellular Mixtures (CIMs)

CIMs from different bacteria were prepared as follows: All cells were individually cultured in a tryptic soy broth (TSB) in a 5 mL volume overnight and an aliquot of each was further fresh cultured until the optical density reached ~1.0 (~10^9^ cells/mL). Then, 1 mL of each bacterial culture was centrifuged at 15000 g for 10 min and the clear supernatant was discarded. The cells were suspended in 500 µL of 1x reaction buffer (HEPES 50 mM, NaCl 150 mM, MgCl_2_ 15 mM, Tween 20 0.01%, pH 7.5). This cell suspension was heated at 90 °C for 5 min and left at room temperature for another 10 min. After centrifugation at 15000 g for 10 min, the clear supernatant was collected and passed through a 0.2 µm filter disc (Acrodisc). This supernatant (corresponds to ~2 × 10^9^ cells/mL) was aliqouted and stored at −20 °C and used in the cleavage experiments when needed.

### DNAzyme Immobilization onto Microzones and Cleavage Test

To identify the best conditions for DNAzyme cleavage, a 100 µL solution of DNAzyme (200 µM, 1 pmol/5 µL) was prepared in four different solutions: 1) 1x RB (50 mM HEPES, 150 mM NaCl, 15 mM, MgCl_2_, 0.01% Tween 20, pH 7.5), 2) in RB including 0.25 M trehalose (TH), 3) in 1x RB including 8% (w/v) pullulan (PL), and 4) in 1x RB including 8% PL and 0.25 M TH. Next, 5 µL of each DNAzyme solution was dispensed onto 6 microzones (3 for controls tested against reaction buffer only and another 3 for testing against undiluted CIM (~10^9^ cells/mL)) and allowed to air dry overnight in the dark. Ten microliters of RB or CIM solutions (~10^7^ cells) was then added to each control or test microzone, respectively. The paper was incubated at room temperature for 30 min and imaged using a Chemidoc^TM^ fluorescence Imager (Bio-Rad). The fluorescence intensity of each microzone was measured and analysed using ImageJ software to produce an intensity value (http://rsb.info.nih.gov/ij/download.html). The cleavage reaction mixtures from each microzone were collected and subjected to 10% denaturing gel electrophoresis (dPAGE). Each gel was scanned for fluorescence using a Chemidoc^TM^ fluorescence Imager (Bio-Rad) with autoexposure settings. The images from the instrument were converted into TIFF images with no further manipulation. Specific sections, denoted by dashed lines in Fig. S2A–D, were used to produce the data shown in Fig. 2 (see Fig. 2 caption for more details).

### Detection of Bacteria on Microzones without Pre-Lysis

Initial studies utilized heating of cells at 90 °C to induce lysis and liberate the intracellular target for analysis. To avoid the use of heating to lyse the cells, which may cause denaturation of a protein-based target, varying amounts of the bacterial lysing agent lysozyme (0, 25, 50, 100, 150 µg) was included in the DNAzyme mixture with pullulan and trehalose. After immobilization and drying, the cleavage test was carried out. Based on the fluorescence signal, 100 µg of lysozyme per microzone was found to be the optimal amount and was used for all subsequent experiments. Next, a mixture of 200 nM DNAzyme (final concentration), 8% pullulan (w/v), 0.25 M trehalose and 1.4 mM lysozyme was prepared and printed on the 96 well paper microzones plate (5 µL in each microzone) using a Biodot printer at a rate of 100 Hz and 1.2 millisecond drop deposition time. The paper was dried and tested for cleavage reactions. A mixture of 200 nM DNAzyme (final concentration), 8% pullulan (w/v), 0.25 M trehalose without lysozyme was also printed as a control to be compared with whole cell and pre-lysed samples. 10 µL of the whole cell sample (~10^7^ cells total) and the pre-lysed sample (equivalent to ~10^7^ cells) was added to the designated microzones (Fig. 3A) and allowed to react for fluorescent signal generation. The fluorescent signal was collected using a Chemidoc^TM^ fluorescent imager at different time points (Fig. 3A) and analyzed by ImageJ Software. The fluorescence values were plotted using Microsoft Excel software.

### Sensitivity Test

To evaluate the sensitivity of the microzone sensor, we followed our previously reported protocol^26^. Briefly, 100 µL of freshly cultured *E. coli* (OD ~1.0) cells was serially diluted 10-fold in 1 mL PBS (phosphate buffer saline) to achieve cell concentrations of 10^0^–10^8^ cfu. 100 µL from 7th dilution was spread onto a tryptic soy agar (TSA) plate and incubated at 37 °C overnight to quantify cell numbers using the cell counting method. This experiment was done in triplicate. Bacterial cells from all tubes were isolated by centrifugation and stored at −20 °C until use. Prior to use, cell pellets in each tube were suspended in 10 µL of 1x reaction buffer and applied to the microzones. The measurement and analysis of the fluorescence signals was done as described earlier.

### Selectivity Test

To evaluate selectivity, 5 mL of each type of cells (*E. coli*, *B. subtilis*, *L. monocytogenes* and *S. typhimourium*) was cultured overnight after which a fresh culture was conducted in a 5 mL volume until the OD reached ~1.0. Cells were isolated from 1 mL of each bacterial culture by centrifugation. Preparation of CIMs (~10^9^ cells/mL) from each of the bacterial cultures, as well as the cleavage reaction and signal analysis were conducted as described above.

### High Throughput Printing Using a Biodot Printer

A bioink solution consisting of 8% pullulan including 200 nM RFD-EC and 0.25 mM trehalose (1 mL volume) was prepared and placed into a glass vial from which sample was aspirated in a Biodot 3060 XYZ printer. The printing parameters were set for 5 µL to be printed per microzone. After printing, the paper was air dried overnight. The Biodot instrument was also used to dispense the sample onto each of the microzones. After 60 min of cleavage reaction the signal was obtained by Chemidoc™ imager, analyzed by ImageJ software and plotted to assess signal reproducibility.

### Detection of *E. coli* in Beverages

The performance of the printed paper sensor was evaluated with *E. coli* spiked into milk, apple juice and drinking water. An overnight culture of *E. coli* was spiked in drinking water (collected from McMaster University campus) to produce a final concentration of 10^7^ cells/mL. 10 μL of this sample was added to the DNAzyme printed paper microzones and allowed to react. A control experiment with the water alone (without *E. coli*) was also performed. After reaction at room temperature for 60 min, the paper was imaged for fluorescence intensity (Chemidoc^(^™^)^ fluoroImager, Bio-Rad). In the case of milk and juice, the background fluorescence was initially tested at dilutions of 2-fold up to 16-fold to produce solutions containing 50%, 25%, 12.5% and 6.25% of milk (2% skimmed milk, Neilson™ brand) and apple juice (100%, Minute Maid™ brand), respectively, in the reaction buffer (Fig. S6). Based on the autofluorescence and pH, 12.5% milk and 25% apple juice were chosen for the DNAzyme performance test, and these samples were spiked with *E. coli* to a final concentration of 10^7^ cells/mL. The cleavage and fluorescence signal was obtained in the same manner as described for drinking water. The results are depicted in Fig. 5.

### Stability test against nuclease

To test the stability of the printed DNAzyme on the paper microzones, a mixture of the DNAzyme was prepared with pullulan including *E. coli* RNase I (0.01 U/µL). The mixture was immediately dispensed on the microzones and air dried. After overnight storage at room temperature, 1x reaction buffer was added on the microzones and allowed to fully dissolve. The paper was imaged for fluorescence as described above. A control experiment with the pullulan and DNAzyme alone without spiking with RNase I was conducted for comparison. The results are presented in Fig. S8 in the electronic supplementary information.

### Data availability

All data generated or analysed during this study are included in this published article (and its Supplementary Information files).

## Electronic supplementary material

A Printed Multicomponent Paper Sensor for Bacterial Detection
