# Supplementary material for: A Printed Multicomponent Paper Sensor for Bacterial Detection
Source: Sci Rep. 2017 Sep 26;7:12335. doi: 10.1038/s41598-017-12549-3 (PMC5615064; doi:10.1038/s41598-017-12549-3)
Supplement: Supplementary file 1 — A Printed Multicomponent Paper Sensor for Bacterial Detection [file 41598_2017_12549_MOESM1_ESM.pdf]

A Printed Multicomponent Paper Sensor for Bacterial Detection

M. Monsur Ali,[1] Christine L. Brown,[2] Sana Jahanshahi-Anbuhi,[2] Balamurali Kannan,[1] Yingfu Li,[3] Carlos D.M. Filipe,[*2] and John D. Brennan*[1]

[1]Biointerfaces Institute, McMaster University, 1280 Main St W, Hamilton, Ontario L8S 4L8, Canada.

[2]Department of Chemical Engineering, McMaster University, 1280 Main St W, Hamilton, Ontario L8S 4L7, Canada.

[3]Department of Biochemistry and Biomedical Sciences, McMaster University, 1280 Main St. W., Hamilton, ON L8S 4K1, Canada

*Email: filipec@mcmaster.ca and brennanj@mcmaster.ca

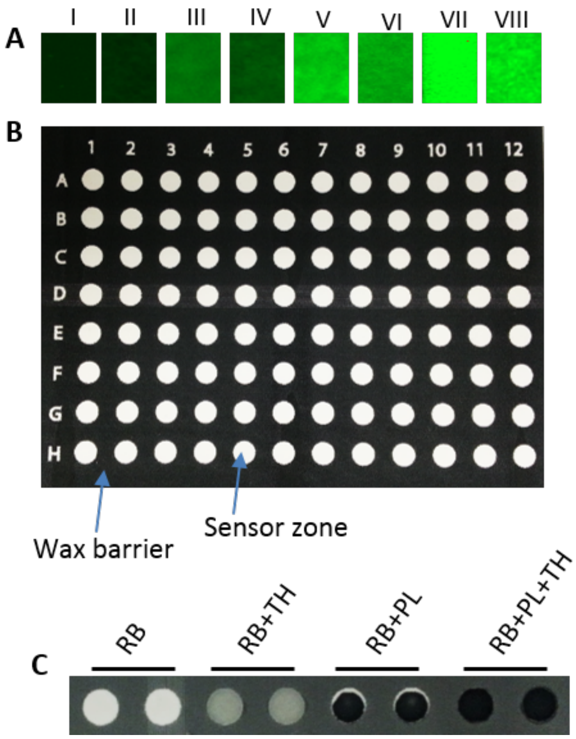

Figure S1 Search for a suitable paper substrate, microzone fabrication and sensor immobilization. A) Background fluorescence of different paper substrates. I) Flexible nitrocellulos paper baked with thin paper layer, II) Whatman#1, III) Whatman#3, IV) Recycle printing paper, V) Whatman#1 coated with plastic in one side, VI) Glossy coated whatman#3, VII) Adsorbent pad and VIII) Food packaging hard paper. B) Microzones fabricated paper after wax printing (black is wax coating which provides hydrophobic barrier, white empty zones are for DNAzyme printing. C) Physical appearance of the microzones after immobilization of DNAzyme probe under different condition and drying (RB: DNAzyme immobilized with reaction buffer, RB+TH: DNAzyme immobilized with RB and trehalose (TH), RB+PL: DNAzyme immobilized with RB and pullulan (PL), and RB+PL+TH: DNAzyme immobilized with RB including PL and TH).

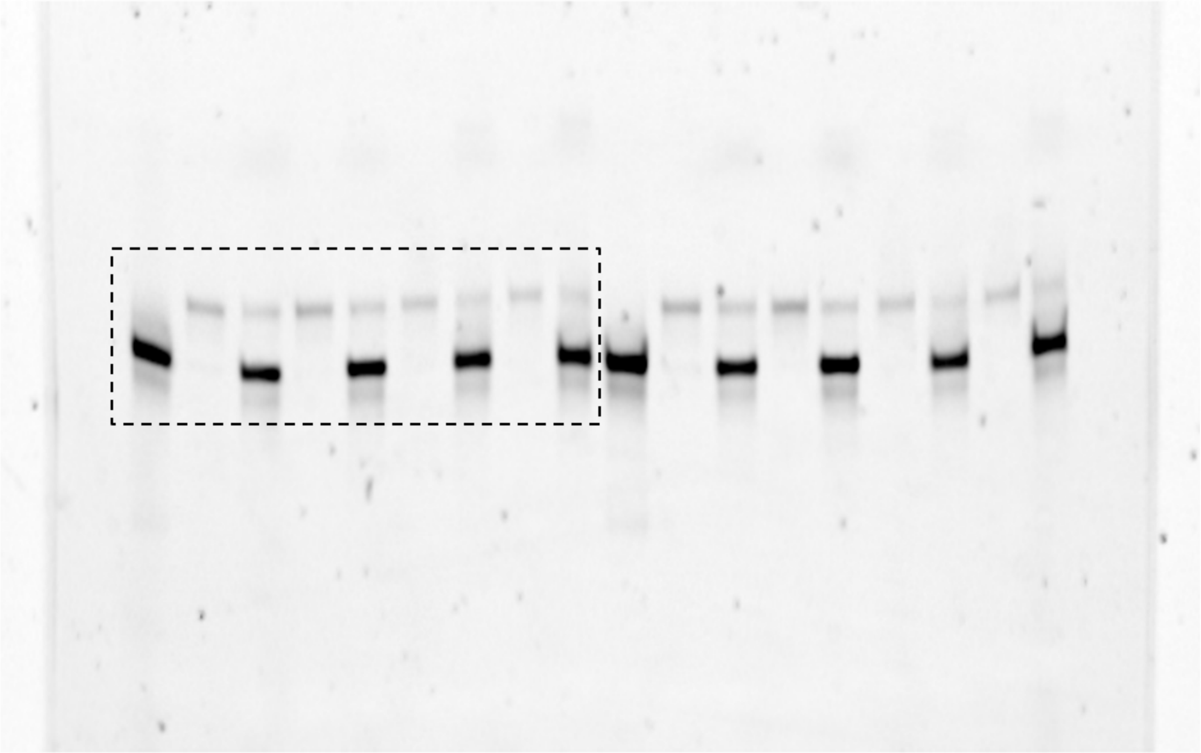

Figure S2A Full size gel image of Figure 2B (in duplicate). Dashed box image was cropped and used in Figure 2B.

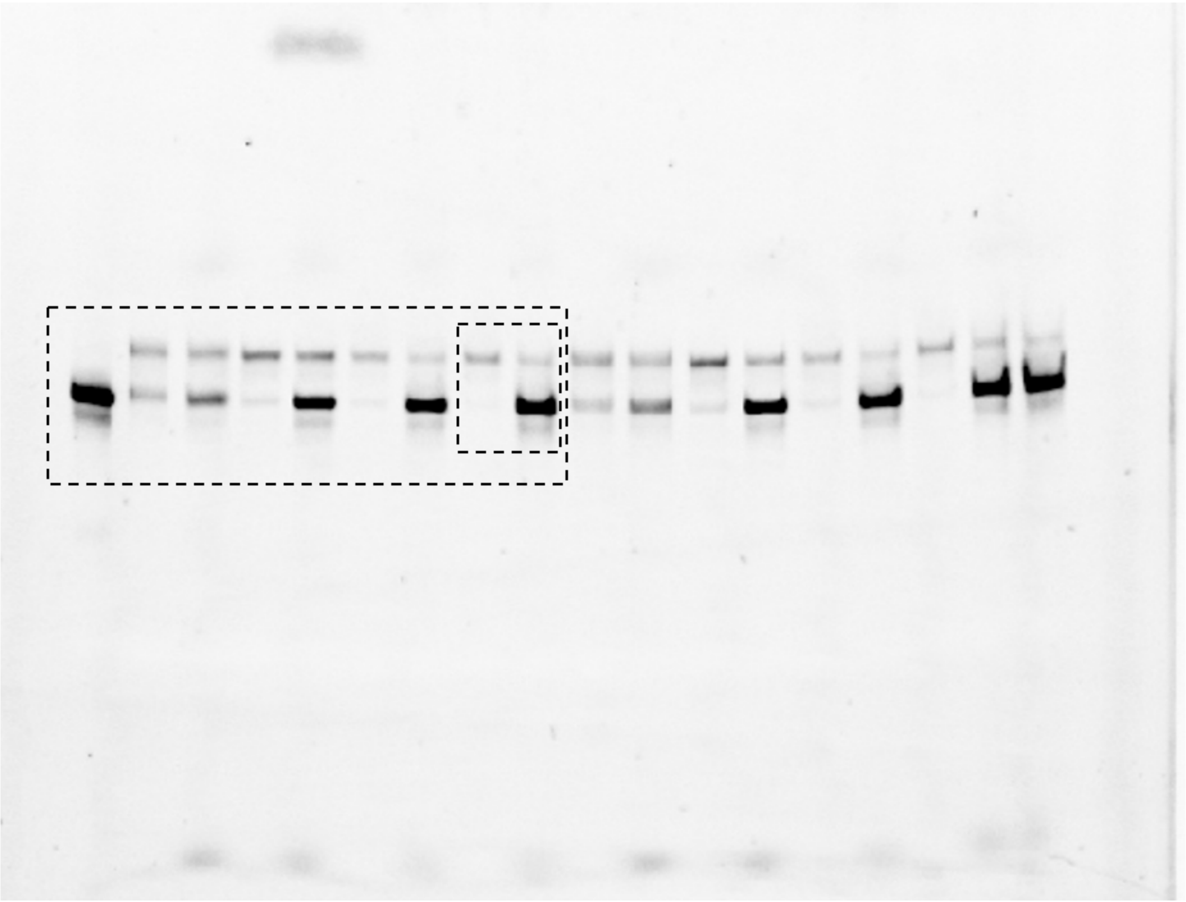

Figure S2B Full size gel image of Figure 2D (in duplicate). Large dashed box image was cropped and used in Figure 2B. Small dashed box image was cropped and used in Figure 2E; labeled as 7 days

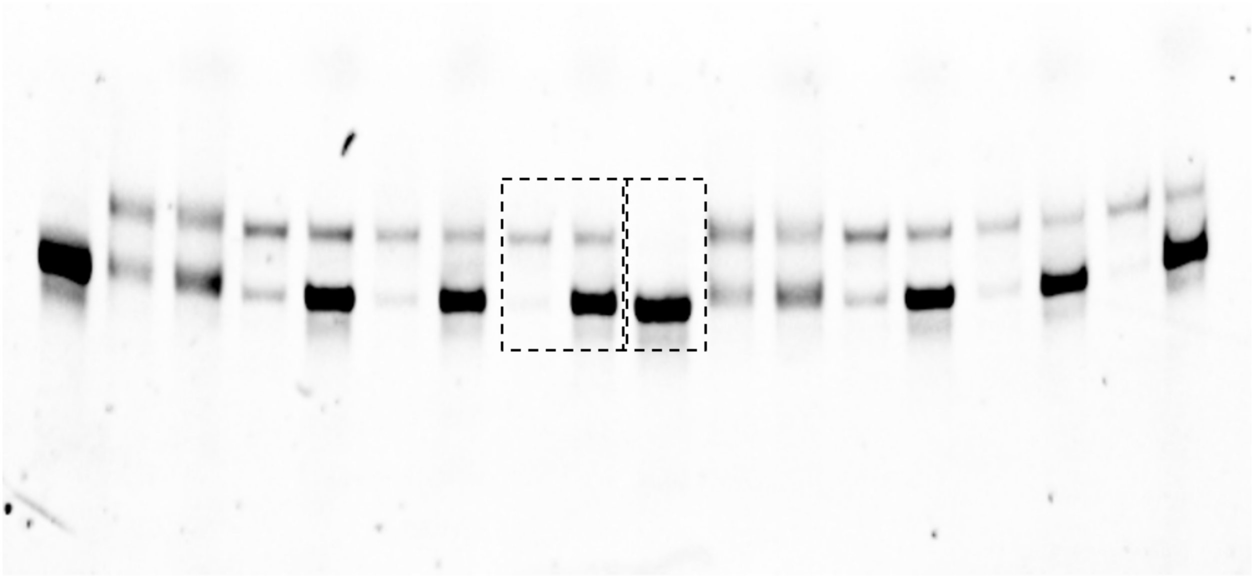

Figure S2C Full size gel image of Figure 2E. Dashed box images were cropped and used in Figure 2E; labeled as 90 days. The right boxed image was taken as marker which was prepared by treating the DNAzyme with alkali and heat at 90 ^{o}C for 10 min.

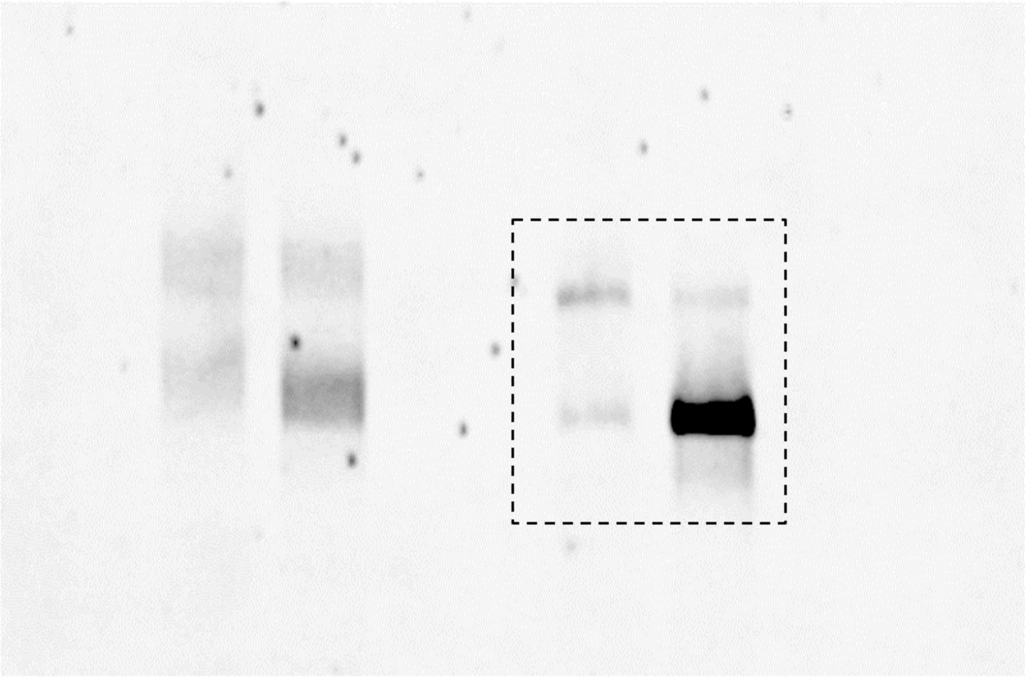

Figure S2D Original gel image of Figure 2E. Dashed box images were cropped and used in Figure 2E; labeled as 180 days (The left image without boxed was for the DNAzyme storage printed with buffer alone excluding pullulan and trehalose).

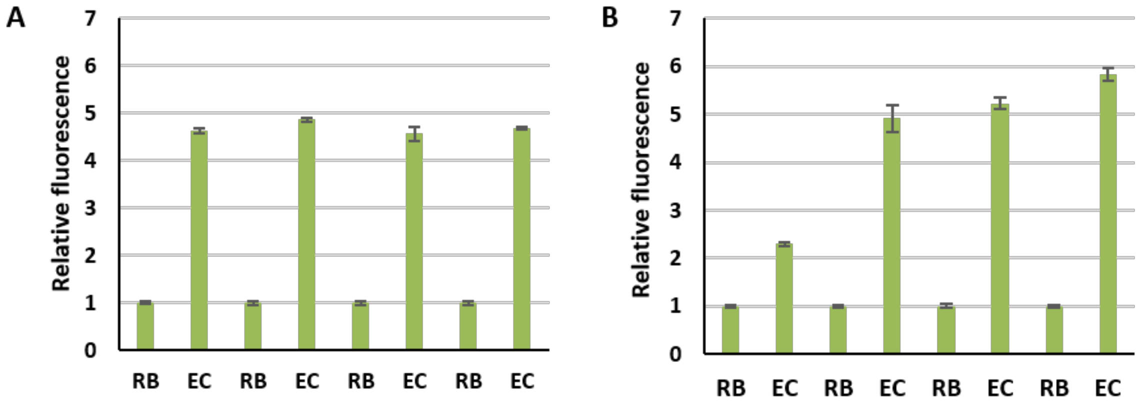

Figure S3 Fluorescence signal calculated from experiments of Figure 2A and 2C in the main text. Error bars are based on triplicate experiments

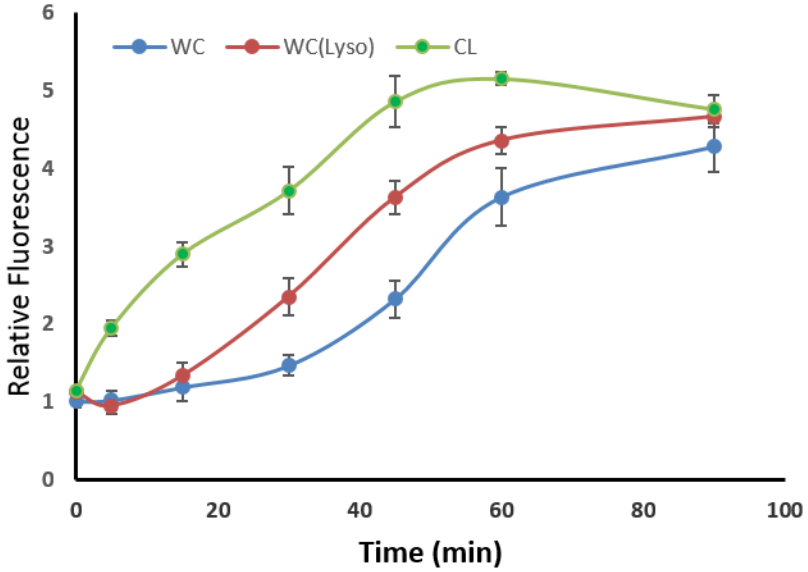

Figure S4 Fluorescence signal in the presence of CL (green), WC in lysozyme/DNAzyme printed microzone (red) and WC in only DNAzyme printed microzone (no lysozyme). Data were calculated from experiments of Figure 3A in the main text. Error bars are based on the standard deviations of triplicate experiments

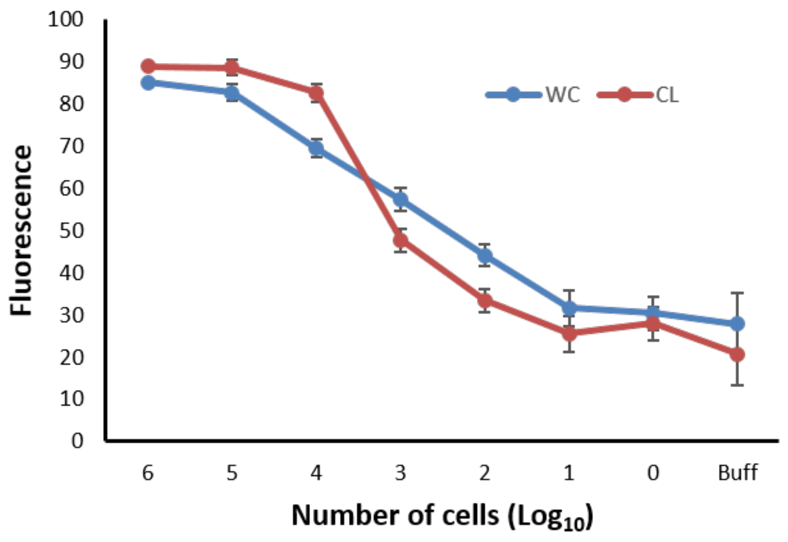

Figure S5 Analyzing the limit-of-detection by pre-lysed cells sample (CL) and whole cells (WC) without lysis. Error bars are based on the standard deviations of triplicate experiments

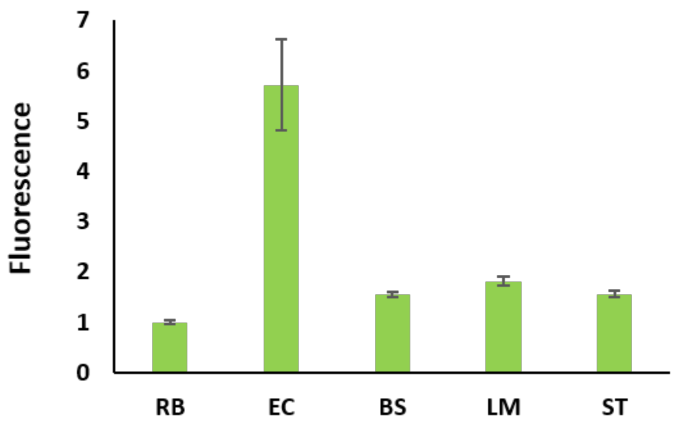

Figure S6 Fluorescence signal calculated from experiments of Figure 3C in the main text. Error bars are based on triplicate experiments

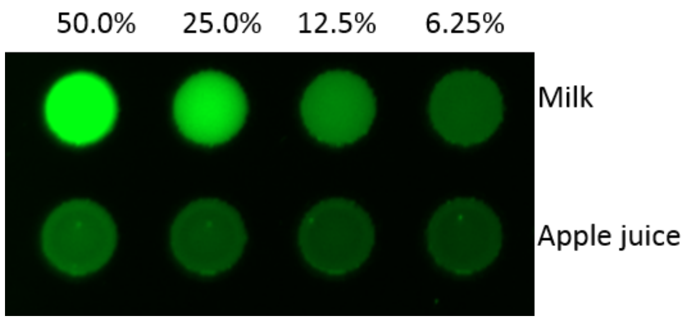

Figure S7 Autofluorescence test of milk and apple juice at different concentrations

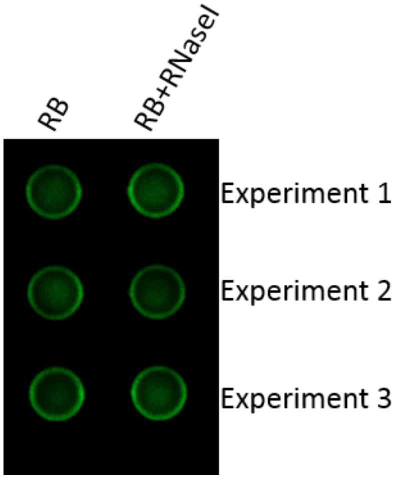

Figure S8 Nuclease degradation test of printed paper sensor.
